# Signal Propagation in Proteins and Relation to Equilibrium Fluctuations

**DOI:** 10.1371/journal.pcbi.0030172

**Published:** 2007-09-21

**Authors:** Chakra Chennubhotla, Ivet Bahar

**Affiliations:** Department of Computational Biology, School of Medicine, University of Pittsburgh, Pittsburgh, Pennsylvania, United States of America; Stanford University, United States of America

## Abstract

Elastic network (EN) models have been widely used in recent years for describing protein dynamics, based on the premise that the motions naturally accessible to native structures are relevant to biological function. We posit that equilibrium motions also determine communication mechanisms inherent to the network architecture. To this end, we explore the stochastics of a discrete-time, discrete-state Markov process of information transfer across the network of residues. We measure the communication abilities of residue pairs in terms of hit and commute times, i.e., the number of steps it takes on an average to send and receive signals. Functionally active residues are found to possess enhanced communication propensities, evidenced by their short hit times. Furthermore, secondary structural elements emerge as efficient mediators of communication. The present findings provide us with insights on the topological basis of communication in proteins and design principles for efficient signal transduction. While hit/commute times are information-theoretic concepts, a central contribution of this work is to rigorously show that they have physical origins directly relevant to the equilibrium fluctuations of residues predicted by EN models.

## Author Summary

In recent years, there has been a surge in the number of studies using network models for understanding biomolecular systems dynamics. Essentially, two different groups of studies have been performed, driven by two different communities. The first is based on molecular biophysics and statistical mechanical concepts. Normal mode analyses using elastic network models lie in this group. The second is based on information theory and spectral graph methods. The present study demonstrates for the first time that signal transduction events directly depend on the fluctuation dynamics of the biomolecular systems, thus establishing the bridge between the (newly proposed) information-theoretic and the (well-established) physically inspired approaches. We have applied the new approach to five different enzymes. Functionally active residues are shown to possess enhanced communication propensities. Furthermore, secondary structural elements emerge as efficient mediators of communication. These results provide us with important insights for protein design and mechanisms of allostery.

## Introduction

Proteins function neither as static entities nor in isolation, under physiological conditions. They are instead subject to constant motions and interactions, both within and between molecules. These motions can be either random fluctuations or concerted functional changes in conformations; and their sizes can vary from localized motions (e.g., single amino acid side chain reorientations) to large-scale global motions (e.g., domain–domain or intersubunit movements). While motions in the nanoseconds regime can be explored by full atomic simulations, understanding those involving large-scale structural rearrangements remains a challenge. In recent years, elastic network (EN) models in conjunction with modal analysis, and in particular the Gaussian Network Model (GNM) [1–3], have been widely used for elucidating the collective dynamics of proteins and exploring their relevance to biological function [4–9].

We posit that these collective motions also determine communication patterns that are inherent to the native architecture. To explore the validity and implications of this concept, we assume a discrete-time, discrete-state Markov process [10–11] of “information” transfer across the network of residues and measure two basic quantities: *hitting time* and *commute time* [11]. Hitting time *H*(*j,i*) is the expected number of steps it takes to send information from residue *v_i_* to residue *v_j_*, and this may not be the same as *H*(*i,j*). Commute time *C*(*i,j*) is by definition the sum: *H*(*i,j*) *+ H*(*j,i*). Hitting time has directionality, while commute time does not.

A major goal in this study is to relate the hitting (and commute) times derived from the Markovian stochastics model to the equilibrium fluctuations (mean-square fluctuations and cross-correlations) of residues predicted by EN models, thus bridging the gap between two disciplines, information theory and statistical mechanics. To this end, using the theory of generalized matrix inverses [12–14], we show that hitting/commute times can be expressed in terms of the Kirchhoff matrix of inter-residue contacts that underlie the GNM methodology. Additionally, we present new insights into the signal transduction properties of enzymes, the catalytic residues of which are shown to be distinguished by their fast and precise communication abilities.

The paper is organized as follows. The Results are divided into three parts: first we present the Markovian stochastic model of information diffusion developed for exploring the inter-residue communication in proteins. The process is controlled by transition probabilities for the passage/flow of information across the nodes, which in turn is based on the internode affinities derived from atom–atom contacts in the folded structures. Second, we describe the evaluation of hit and commute times, and illustrate these concepts by presenting the application of the methodology to five different enzymes. Strikingly, active residues are distinguished by their effective communication stochastics. Third, we present a rigorous derivation of the mathematical relation (Equation 15) between inter-residue hit/commute times, and their fluctuation dynamics derived from purely statistical mechanical theory. This important relation establishes the bridge between information-theoretic quantities evaluated here for proteins and the intrinsic structural dynamics of proteins as described by physics-based models, and provides a new avenue for further examination of protein allostery using the new information-theoretic perspective.

## Results

### Information-Theoretic Description of Network Communication

#### Affinity matrices.

The protein structure is modeled as a network of *n* nodes, each representative of a given residue *v_i_*, for 1 < *i* < *n*. The interaction strength, or the *affinity a_ij_*, between residues *v_i_* and *v_j_* is defined as

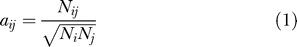
where *N_ij_* is the total number of *atom–atom* contacts made between residues *v_i_* and *v_j_* based on a cutoff distance of *r*_c_ = 4 Å and (*N_i_*, *N_j_*) are the total numbers of heavy atoms in the individual residues (*v_i_*, *v_j_*). The affinity matrix is very similar to a contact map or the adjacency matrix of a graph. Non-zero entries in the adjacency matrix denote if two residues are in contact, while the entries in the affinity matrix scale with the number of atom–atom contacts between the residue pair. This representation captures to a first approximation the strong (weak) interactions expected to arise between residue pairs with large (small) number of atom–atom contacts. The denominator corrects for the biases induced by size effects, e.g., for the larger number of atom–atom contacts inherently made by larger size amino acids, thus permitting us to assign affinities purely based on differential interactions. Note that a similar expression has been adopted by Brinda and Vishveshwara [15].

The affinities provide a measure of the local interaction density *d_j_* at each residue *v_j_* as *d_j_ =* 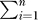 *a_ij_*. Note that we can define an equivalent mass-spring system having *stiffness matrix γΓ* where *Γ* is defined in terms of the affinity and degree matrices, ***A*** = { *a_ij_* } and***D*** = diag{*d_j_* } respectively, as

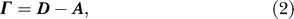
and *γ* is a force constant uniform over all springs.***Γ*** is also called the Kirchhoff matrix or the *combinatorial Laplacian* in graph theory [16].

#### Markov model of network communication.

A *discrete-time, discrete-state* Markov process [11,16] is defined by setting the communication probability between residue pairs to be a function of their affinity. In particular, we define

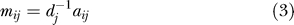
as the conditional probability of transmitting information to residue *v_i_* in *one time step* given that the signal is initially positioned at residue *v_j_*. Note, *d_j_* serves as a normalizing factor to ensure

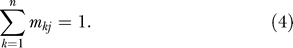
The conditional probability matrix ***M*** = {*m_ij_*}, also called the Markov transition matrix, defines the stochastics of information diffusion over the network of residues, via

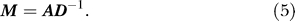



Suppose the probability of initiating the Markov propagation process at node *j* is *p_j_*(0). Then, the probability of reaching residue *v_i_* in one time step is *m_ij_p_j_*(0). In matrix notation, the probability of ending up on any of the residues ***v*** = [*v_1_, v_2_,...,v_n_*] after one time step is given by the distribution ***p***(1) = ***M*** ***p***(0). Or, after *k* steps,

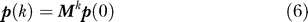
where ***p***(*k*) = [*p*_1_(*k*),...,*p_n_*(*k*)] represents the *n*-dimensional vector of the probabilities of residing at node *i* (1 ≤ *i* ≤ *n*) at step *k*.

Assume there is a path connecting every pair of residues in the network. Then, as the number of steps *β* approaches infinity, ***p***(*β*) approaches a unique *stationary* distribution ***π*** = [*π*_1_,*π_2_*,...,*π_n_*], the elements of which are given by

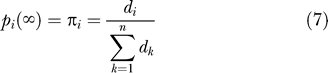
Whereas the evolution of the diffusion process is a function of the starting distribution, the stationary distribution is invariant to the details of initiation.

In the continuous time limit [17], the change in probabilities follows the master equation 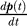 = (***M*** − ***I***) ***p***(t) with a solution ***p***(t) = exp[(***M*** − ***I***)t] ***p***(0), where ***I*** is an identity matrix of dimension *n* × *n*. Here,***M*** − ***I*** replaces the transition rate matrix, assuming the time elapsed between successive jumps obeys an exponential probability distribution [17]. Note that (i) in this limit, t replaces *k*δt where δt is the mean step size implicitly used in the discrete approximation, and (ii) the stationary distribution corresponding to the continuous process is identical to that obtained for the discrete process such that the detailed balance *m_ij_π_j__=_ m_ji_π_i_* holds.

#### Hit/commute times as a function of Markov transition probabilities.

The hitting time *H*(*j,i*) is the average number of steps it takes for the information residing at residue *v_i_* to be transmitted to residue *v_j_* for the *first* time. We will term residue *v_i_* a *broadcaster* and residue *v_j_* a *receiver*.

The calculation of *H*(*j,i*) requires the consideration of all possible pathways on the network, each being weighted by the product of transition probabilities along the path, starting from *v_i_* and ending at *v_j_*. An efficient recursive formula can be derived for the calculation of *H*(*j,i*) as follows. Suppose the passage from *v_i_* to *v_j_* is performed in two stages, from *v_i_* to a neighbor *v_k_* that is one step away, succeeded by probabilistic passages from *v_k_* to the final destination *v_j_*. Furthermore, assume we know the hit time *H*(*j,k*) from the intermediate node *v_k_* to the destination node *v_j_*. Summing over all the intermediate nodes, the hitting time from *v_i_* to *v_j_* is simply

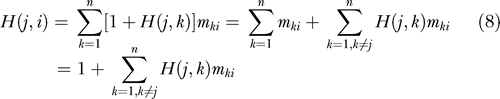
where Equation 4 is used on the first term on the right hand side. By definition, *H*(*i,i*) *=* 0. Equation 8 provides a self-consistent method for evaluating the hitting time between any two nodes.

The commute time is defined by the sum of the hitting times in both directions, i.e.,

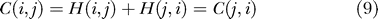



Note that the commute time is symmetric by definition while *H*(*i,j*) is not, as will be illustrated below for example proteins. See the section “Pedagogical example to compute hit/com-mute time” in Methods for hit time analysis of a simple network.

In the calculations below, it proves convenient to define the average hitting times in both directions, as well as the average commute time, for each individual residue as

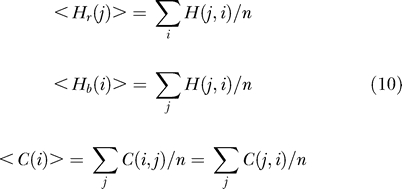



<*H_r_*(*j*)> and <*H_b_*(*i*)> provide a measure of the respective receiver and broadcasting properties of residues *j* and *i*, and <*C*(*i*)> provides a measure of signal transduction properties, in general. Commute time is also known by the name of “resistance distance” in computational chemistry [14,18].

### Application to Enzymes

#### Mapping of hit/commute times between all residue pairs.

Figure 1A displays the hitting times *H*(*j,i*) computed for all residue pairs (*v_i,_ v_j_*) for an example enzyme, snake phospholipase A2 (Protein Data Bank (PDB) [19], 1bk9 [20]). The blue regions correspond to short hit times, and red regions to long hit times, as indicated by the scales on the right. The map consists of the elements of the hitting time matrix ***H***. Accordingly, the *j* ^th^ row indicates the number of steps required for a signal to hit residue *v_j_*, starting from any residue *v_i_*. The values are relatively uniform within each row, which reveals that no single residue stands out as an efficient broadcaster, i.e., <*H_b_*(*i*)> = 340.3 ± 1.5 for all *i*. On the other hand, comparing different rows we note that some residues are much better receivers than others. <*H_r_*(*j*)> values indeed vary over a broader range of 340.3 ± 124.8.

**Figure 1:**
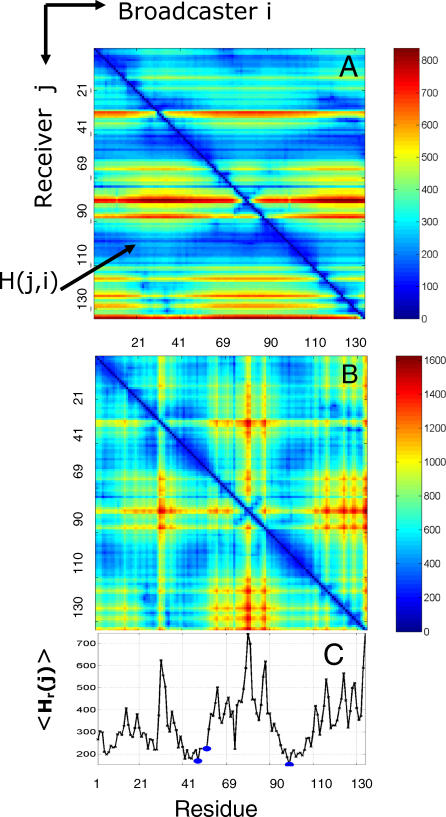
Distribution of Hitting Times and Commute Times for Phospholipase A2 (1bk9 [20]) (A) Hitting time *H*(*j,i*) distribution shows more variation between rows than between columns, indicating that residues differ in their ability to receive signals, while their broadcasting properties are more uniform. (B) Shows commute time *C*(*i,j*) distribution. (C) Displays the average hitting times evaluated from (A). All three catalytic residues (blue dots) exhibit short hitting times.

The higher ability of particular residues to transduce signals is also reflected in the commute times displayed in Figure 1B. The commute time is symmetric by definition, but the hitting time is not. The blue regions along the diagonal show that there is efficient communication along sequential residues, although we also observe several sequentially *distant* residue pairs that efficiently communicate. While the majority of these residue pairs are spatially close, as will be shown below, there is not necessarily a one-to-one correspondence between commute times and spatial distances, and some residue pairs emerge as more efficient communicators than others despite their longer physical separation.

#### Communication properties of individual residues.

Figure 1C displays the mean hitting time <*H_r_*(*j*)> for each residue. Minima in this curve point to residues that are effective in receiving signals. It is worth mentioning that the mean commute time <*C*(*i)*>, which involves both receive and broadcast times, will have the same profile shape as the mean hitting time, because the broadcasting ability is roughly the same for all residues (as observed in Figure 1A).

#### Catalytic residues distinguished by fast and precise communication.

It is of interest to examine the signal transduction properties of catalytic residues. Phospholipase A2 has three catalytic residues: His48, Tyr52, and Asp99. Notably, all three residues (indicated by blue dots) are found to be located in minima (Figure 1C), i.e., the effective time required for these residues to establish communication with others is minimal.

To additionally highlight the enhanced communication properties of the catalytic residues, we plot in Figure 2 the mean <*H_r_*(*j*)> and standard deviations *σ*(*H_r_*(*j*)) for all residues. The catalytic sites are marked as red crosses. We observe that their hitting times (as well as their commute times) are *short* (in terms of their mean values) and *precise* (in terms of their standard deviations). From the plot, we also observe that for the same mean hitting time, the precision can vary by several folds. Figure 2 also displays the ligand-binding residues by black + symbols. While ligand-binding residues also exhibit relatively short hit times and small variance, they do not appear to be as distinctive as the catalytic residues.

**Figure 2:**
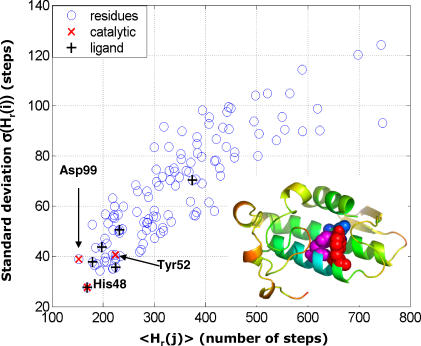
Average Hit Times < *H_r_*(*j*) > versus Their Standard Deviations *σ*(*H_r_*(*j*)) Catalytic residues (red crosses) are fast and precise, being located at the lower left end of the plot. Ligand-binding residues are indicated by black +.

Figure 3 illustrates similar results for four other enzymes: HIV-1 protease [21], ricin [22], human rhinovirus 3C protease [23], and endo-1,4-xylanase [24] (see caption for more details). The catalytic residues (highlighted as red dots) exhibit relatively short and narrowly distributed hitting times in each case. Ligand-binding residues (blue dots), on the other hand, display a wider range of hitting times and deviations, consistent with the results for phospholipase A2. At least one of the catalytic residues, indicated by the label, is distinguished in each case by its high communication speed and precision.

**Figure 3:**
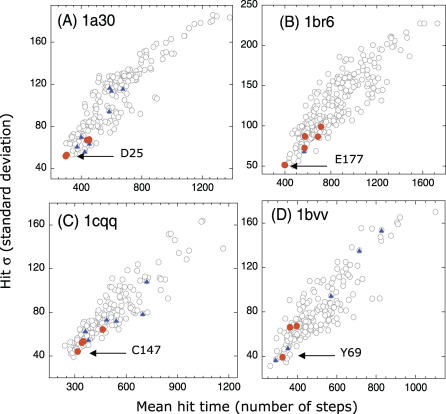
Results from Hitting Time Analysis for Four Enzymes (A) HIV-1 protease (1a30, [21]), (B) Ricin (1br6, [22]), (C) Human rhinovirus 3C protease (1cqq, [23]), and (D) Endo-1,4-xylanase (1bvv, [24]). The plots reveal the tendency of catalytic residues (D25 and D30 in (A), Y80, V81, G121, Y123, E177, and R180 in (B), H40, E71, G145, and C147 in (C), and Y69, E78, and E172 in (D); red dots) to exhibit fast and precise communication, in accord with the results for phospholipase A2 (Figure 2). Ligand-binding residues are shown by blue dots. The catalytic residues with the highest communication propensity are labeled.

### Bridging Information-Theoretic Concepts and Physically Inspired Models

#### Fluctuations determine communication.

Consider the hitting time to the *n* ^th^ residue *v_n_* starting from residue *v_i_*. Using Equation 8 and substituting *n* for *j*, we can use a truncated version of the Markov transition matrix 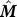 where the *n* ^th^ row and column are deleted to obtain

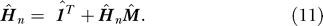



Here, 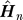 denotes *n* ^th^ row of the hitting time matrix ***H*** truncated to the first *n −* 1 elements and 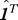 is a row vector of length (*n −* 1) of all 1′s. 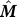 can be expressed in terms of a similarly truncated Kirchhoff matrix 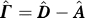 using Equations 2 and 5, leading to

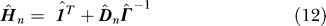
or, in component form,

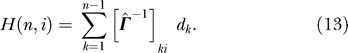



As derived in Methods, 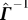 can be expressed in terms of the pseudo-inverse 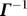 using the theory of generalized matrix inverses [12–14], to obtain

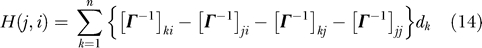
for the hitting time from residue *v_i_* to any arbitrary residue *v_j_*. Substituting from Equation 19 in Methods, where the elements of the inverse of the Kirchhoff matrix are related to residue fluctuations [25,26], we obtain

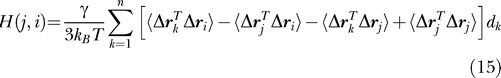
The above equation constitutes the most important result from the present study: it provides the physical basis for the hitting times obtained with the information-theoretic methodology by relating them to correlations between residue fluctuations derived from statistical mechanical theory [25,26]. The meaning of Equation 15 will be further elaborated below upon assessment of the contribution of each term in brackets.

Substitution of Equation 14 in Equation 9 yields an expression for the commute time in terms of *Γ* ^−1^,

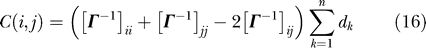
which, using Equation 19, reduces to

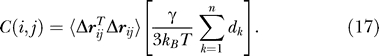
This is our final expression bridging commute times with fluctuations 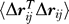 in inter-residue distances. Note that the term in parentheses is a constant for all pairs of residues. Thus, the commute time between residues *v_i_* and *v_j_* is directly proportional to the fluctuations in the distance between these two residues, larger fluctuations entailing longer commute times, and vice versa.

#### More on the physical meaning of hitting times.

The hitting time expression Equation 14 involves three different types of contributions: a one-body term that depends on the destination node, 
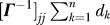
 ; a two-body term that depends on the initial and final nodes, 
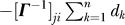
 ; and a series of three-body terms that depend on intermediate nodes, in addition to the two end points, 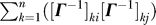 *d_k_*. Of interest is to understand the relative contributions of these three terms. Note that the first is always positive, and increases with the size of destination residue fluctuations; the second may be positive or negative, and the negative sign in front of this term implies that positively correlated residue pairs shorten the hitting time. Likewise, the third term may be positive or negative.

Figure 4 shows the results for phospholipase A2. Figure 4A–4C corresponds to the respective one-body, two-body, and three-body contributions. Note that Figure 4A–4C has different scales, for clearer visualization. As we demonstrate in Figure 4A, the one-body term plays by far a dominant role in determining the resulting hitting times (shown in Figure 1A), i.e., the mean-square fluctuations of the destination node largely determine the hitting time. Residues subject to large amplitude fluctuations require a longer time to be hit, while those subject to small amplitude fluctuations, usually confined to the core or high-density regions, display short hitting times.

**Figure 4:**
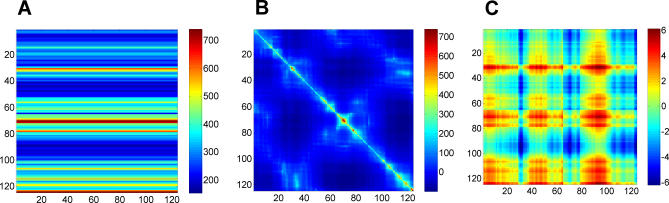
Physical Meaning of Hitting Times Decomposing the hitting time *H*(*j,i*) matrix from Figure 1A into (A) one-body, (B) two-body, and (C) three-body terms, such that summation of these three matrices will reproduce the matrix in Figure 1A. The one-body term involves the fluctuations of only the destination node, apparent by the horizontal stripes seen (A). From the scale of this plot, it is easy to infer that the one-body term dominates the overall computation of the hitting time. However, the source node can modulate the hitting time to the destination node depending on the cross-correlations between the fluctuations of the two nodes (B). (C) reveals the contribution from the three-body terms to be negligibly small.

The two-body term may be positive or negative, depending on the type of cross-correlations between residues *v_i_* and *v_j_.* The negative sign on the two-body term implies that the hitting time is reduced if a residue pair undergoes positively correlated fluctuations (Figure 4B). Anticorrelated residues ([***Γ*** ^−1^]*_ji_* < 0), on the other hand, make a positive contribution to Equation 14, thus increasing the communication time. Finally, from Figure 4C we observe that the contribution from the three-body term is negligibly small.

The qualitative features observed here were verified to be valid for all examined proteins: mainly, the mean-square fluctuations of the destination node play a dominant role in determining the hitting (or commute) time, and the cross-correlations between the two end points may increase or decrease the hit/commute time, depending on the type of correlation. Anticorrelations have a retarding effect, while positive correlations reduce the hitting time. In the extreme case of the two nodes moving in phase, by the same amplitude, the effective hit/commute time approaches zero.

## Discussion

### Communication Distances versus Physical Distances

The commute times provide us with a means of estimating effective communication distances 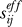 between residues *v_i_* and *v_j_*, using the simple relation

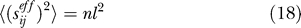
for the mean-square distance traveled by a random walk of *n* steps, with *l* being the average step size. In our case, *l* can be readily estimated from the average distance between connected nodes in the network. For phospholipase A2, *l* is evaluated to be 3.41 Å. Note that this is shorter than the distance (∼3.81 Å) between consecutive α-carbons, because side chain atoms between neighboring residues may get closer to each other. The number of steps, on the other hand, is directly given by the hitting times themselves (as hitting times are expressed in terms of number of steps). For simplicity, we will use *n =* ½*C*(*i,j*) for the effective number of steps for communication between residues *v_i_* and *v_j._*. The average commute time is similar to the recently proposed “diffusion distance” metric for graphs introduced in previous studies [27,28]. The diffusion distance is based on the eigenvalue decomposition of the Markov transition matrix, whereas the hit/commute times are derived from the graph Laplacian.

### How Do Effective Communication Distances Correlate with Physical Distances?

Figure 5A displays the results for phospholipase A2. The effective distance 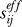 (ordinate) is plotted therein against the physical distance 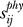 directly evaluated from the PDB coordinates, averaged out over all atoms of residues *v_i_* and *v_j_*. As expected, the effective communication distances increase with physical distance; however, we can also see a broad variability. The points colored red refer to pairs involving the catalytic residue His48.

**Figure 5:**
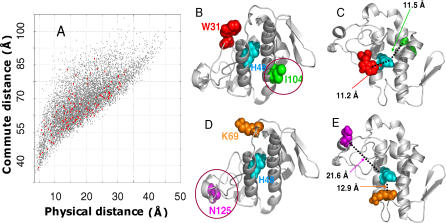
Correlation of Effective Communication Distances with Physical Distances (A) Comparison of efficient communication distances (ordinate) and physical distances (abscissa) for all residue pairs in phospholipase A2. The points colored red refer to pairs involving the catalytic residue His48. (B) and (C) illustrate the differences in communication times, for residue pairs separated by similar distances, and the opposite situation of comparable communication times despite significant differences in inter-residue distances, (D) and (E). See text for more details.

Figure 5B and 5C displays from two different perspectives, two residues (Trp31 and Ile104) located at the same physical distance (11.35 ± 0.15 Å ) from His48, but differing in their communication distances (69Å and 46.5Å) by a factor of approximately 1.5, pointing to the importance of the particular topology, or secondary structural elements, in increasing the effectiveness of communication. The communication with Trp31, located on a loop, turns out to be much slower, in this case. Figure 5D and 5E illustrates the opposite case of two residues (Lys69 and Asn125) that display comparable communication distances (60.5 ± 1.52 Å), while their respective physical distances (12.9 and 21.6 Å) differ by a factor of 1.7, approximately.

### Effect of Secondary Structure

The comparison of the effective and actual (physical) communication distances in Figure 5 suggests that secondary structural elements possess higher abilities in processing signals. To test the validity of this conjecture, we analyzed the distributions of hitting times *H*(*i,j*) to residue *i*, for the three cases where residue *i* is *α*-helical, *β*-strand, or coiled/disordered*.* Figure 6 displays the distributions obtained by combining the results for the examined enzymes. Because the average hitting time increases linearly with the size of a given enzyme, the results for each enzyme are normalized with respect to the number of residues N in each protein, before combining the data. *α*-helical residues are observed to be the most efficient communicators, succeeded by *β*-strand residues (intermediate behavior), while the coiled residues are slower and exhibit a broader distribution of hitting times. Examination of the individual proteins, on the other hand, reveals that *β*-strands may exhibit strong dependence on their spatial location in the 3-D structure of the proteins (Figure 7).

**Figure 6:**
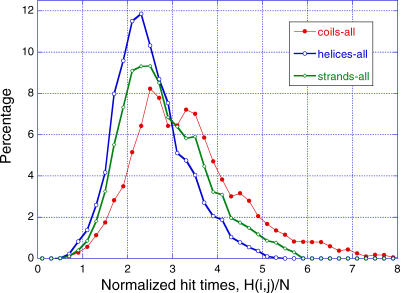
Importance of Secondary Structure in Defining Effective Means of Communication Probability distribution of hitting times *H*(*j,i*) for the cases where residue *j* is located on (A) α-helices, (B) *β*-strands, and (C) loops or disordered regions. A total set of 49,929, 64,732, and 79,444 pairs contribute to the three respective curves, derived from the examined five enzymes. The abscissa represents the hitting time divided by the number of residues, which permits a normalization of the data collected for different proteins. The histograms are based on bins of size 0.2 in the interval [0, 10].

**Figure 7:**
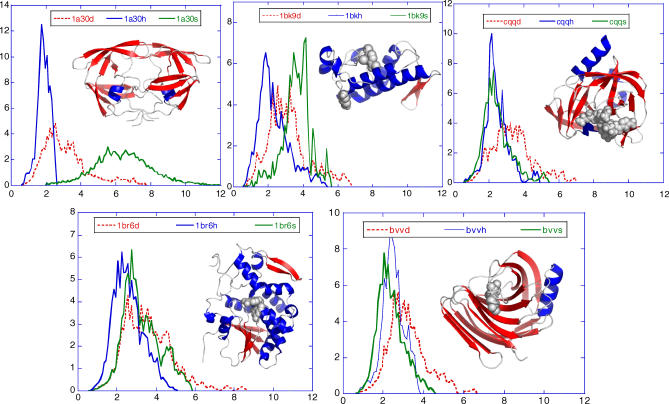
Effect of Secondary Structure on the Hitting Time Distributions for Individual Proteins, Each Shown in a Separate Panel The ribbon diagrams are colored by the secondary structure, namely helices (red), strands (blue), and coils/disordered regions (white). For each enzyme, the probability distribution of hitting times *H*(*j,i*), where *j* is located on (A) α-helices, (B) *β*-strands, and (C) loops or disordered regions is shown in blue, red, and green respectively. The distributions from α-helices and loops/disordered regions have roughly the same shape as the ones shown in Figure 6. However, the distributions for the *β*-strands exhibit significant variations, pointing to a dependency on their spatial location in the 3-D structure of the proteins.

As noted above, the mean-square fluctuations of the destination node play a dominant role in determining the hitting (or commute) time. The higher communication propensity of *α*-helical, and to some extent *β*-strand residues may thus be rationalized by the smaller fluctuations of secondary structural elements compared with coiled regions commonly observed in proteins. It is worth noting, however, that these observations hold for single domain proteins. As illustrated in Figure 8 for a multidomain protein, adenylate kinase, the communication between residue pairs belonging to different domains are usually slower than that between pairs in the same domain.

**Figure 8:**
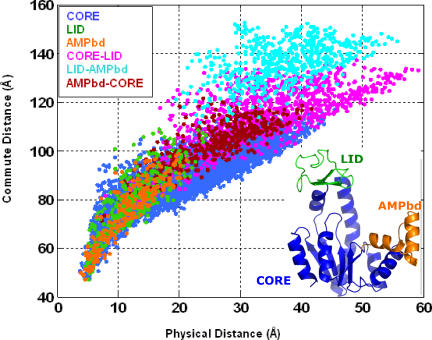
Comparison of Effective (Commute) Distances and Physical Distances between Residue Pairs in E. coli Adenylate Kinase (PDB: 4ake) Effective (ordinate) and physical (abscissa) distances between residues in the CORE, LID, and AMPbd domains (see inset), grouped as intradomain and interdomain distances and shown in different colors for each group. Note that communication between residues in the same domain is more efficient than that between residues in two different domains. This is evidenced by the longer commute distance corresponding to interdomain pairing for a given physical distance, compared with that of intradomain pairs. The inset gives a schematic overview of the distance distributions for intradomain and interdomain pairings.

### Connecting Physically Inspired and Information Theoretic Approaches

Methods based on network models significantly helped in recent years in providing a comprehensible description of the dynamics of biomolecular systems. On the one hand, methods based on fundamental statistical mechanical principles have been proposed for delineating the collective motions of biomolecules [1–9]; on the other, those based on spectral graph theory and machine learning algorithms have been developed for exploring allosteric effects and response to perturbations/mutations in complex structures [29,30]. While these two methodologies concur in their objectives—understanding the complex machinery of biomolecular systems, the connection between these two approaches has been elusive due to their different originating disciplines, as well as the basic quantities they shed light onto: frequency spectrum and normal mode shapes in the former, shortest paths of communication, and hitting/commute times in the latter.

The present study offers a rigorous way of connecting the two approaches, by demonstrating that the commute times between residues *v_i_* and *v_j_*, derived from Markov propagation formalism, directly scale with the mean-square fluctuations 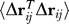 in inter-residue distances (Equation 17). Alternatively, the hitting times are expressed in terms of the elements of the covariance matrix (Equation 15). The proportionality between commute times/distances and fluctuations in inter-residue distances explains the “intriguing balance” (or high correlation) recently reported between shortest path lengths and residue fluctuations [31,32]. What the hit-time/commute-time metrics show is the predisposition of the network to exhibit particular communication patterns. These metrics point to how information diffuses in the system. For a protein that behaves as an elastic body, these communication patterns are expected to be intimately connected to equilibrium dynamics, and the present approach connects the equilibrium fluctuations to the kinetic perspective of diffusion on the graph. This analysis may be viewed as a first step toward building analytical models for elucidating the pathways of energy flow in complex biomolecular systems, complementing ongoing MD efforts along the same lines [33].

### Biological Implications and Future Prospects

Notably, the application to example enzymes point to the more efficient communication propensity and precision of catalytic sites (Figures 1–3), to the role of residue fluctuations and their correlations in transmitting information (e.g., delaying effect of anticorrelated pairs), to the structure-encoded differences in the communication abilities of residue pairs, irrespective of their physical distances; and to the importance of both tertiary contact topology and local (secondary) structure in defining effective means of communication (Figures 5 and 6). Also, irrespective of physical distance, interdomain communication tends to be slower than intradomain, as illustrated in Figure 8 for adenylate kinase.

The major advantage of the present stochastic model over the GNM is the fact that the new methodology lends itself to a comprehensive assessment of the communication paths and their efficiency in biomolecular structures. As such, it holds promise for identifying allosteric communication pathways as well as the sites distinguished by high allosteric potentials, thus providing insights into the design principles of biomolecular machines. The presently observed enhancement in the information transfer properties of catalytic residues and secondary structural elements suggests possible design requirements for efficient enzymatic activity. In this context, it is worth noting the relevant studies by Choe and Sun [34] and Maritan and coworkers [35], which point to the dependence of equilibrium dynamics on secondary structural content/type. It remains to be understood whether such special communication abilities of catalytic residues result from their local packing topology or more global features conferred by evolutionary pressure.

We note that finding suitable experimental setup for probing hit-times is a challenge. In general, the residues/interactions involved in information flow, or the changes in inter-residue distances (which directly define the commute times) may be assessed by site-directed mutagenesis and cross-linking experiments as well as spectroscopic methods such as site-directed fluorescence labeling [36] or FRET [37].

Finally, establishing the bridge between these two disciplines will permit us to translate the wealth of concepts and methods developed in information-theoretic approaches, to exploring the signal transduction mechanisms in complex biomolecular systems, thus complementing physically inspired models and methods.

## Methods

### Positional fluctuations based on the GNM***.***

Let Δ***r*** *_i_* and Δ***r*** *_j_* be the fluctuation vectors from the mean locations of residues v*_i_* and v*_j_*. According to the GNM, the cross-correlations between the fluctuations scale as [1]

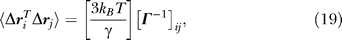
where *k_B_* is the Boltzmann constant and 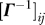 denotes the *ij* ^th^ element of the inverse Kirchhoff matrix ***Γ*** ^−1^. The individual residue mean-square (ms) fluctuations are obtained by substituting *i = j* in Equation 3. Equation 19 is based on purely statistical mechanical considerations, originally put forward by Flory and coworkers for polymer networks [25,26].

### Fluctuations in inter-residue distances.

The mean-square fluctuations 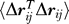 in inter-residue distances Δ***r*** *_ij_* = Δ***r*** *_j_* − Δ***r*** *_i_* can be expressed in terms of the fluctuations in position vectors as

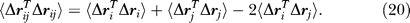

Equation 20 can be rewritten, using Equation 19, in terms of the elements of ***Γ*** ^−1^ as

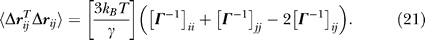
By definition, ***Γ*** is positive semi-definite, i.e., ***Γ*** has rank *n* − 1, so it cannot be inverted. Instead, its pseudo-inverse is computed after eliminating the contribution from the zero eigenvalue.

### Pedagogical example to compute hit/commute time.

Consider a small undirected network of three nodes connected as in

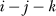
Assume that *a_ij_* = *a_jk_* = 1 and hence the degrees *d_i_* = *d_k_* = 1; *d_j_* = 2. For a random walk initiated at *i* (or *k*), it is obvious that it takes just one time step to reach *j* as *m_ji_* = *m_jk_* = 1. So the hitting time *H*(*j*,*i*) = 1. Similarly, one might be tempted to conclude that the expected number of time steps to reach *k* from *i* is 2. However, this is not the case because once the random walk reaches *j,* there is an equal chance of returning to *i* or going to *k*, because *m_ij_* = *m_kj_* = ½. This recursive argument can be unrolled in two ways. First, using Equation 8, the hit time *H*(*k,i*) can be expressed as

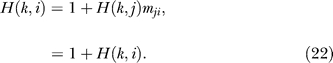
Similarly,

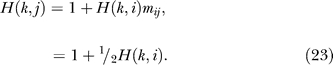



Simultaneous solution of Equations 22 and 23 yields *H*(*k,i*) *=* 4 and *H*(*k, j*) *=* 3. The second way to unroll the recursion is to enumerate the paths between pairs of nodes, as shown in Table 1. The enumeration leads to the calculation of expected time steps

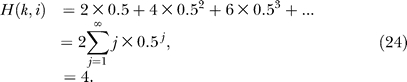



**Table 1:**
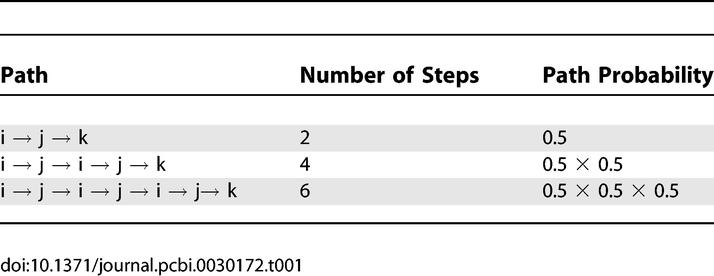
Enumerating *Some* of the Paths Connecting Node *i* with Node *k*

Given the symmetry in the network here, the hitting time *H*(*k,i*) *= H*(*i,k*)*,* but this may not be true in general. To conclude this example, consider the hitting time from *j* to *i*. Again, the random walk unrolled partially is shown in Table 2. This enumeration leads to

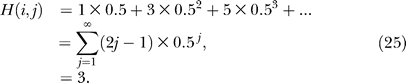
Clearly, the hitting time *H*(*j*,*i*) ≠ *H*(*i*,*j*). While iterative methods, using Equation 8, are one way to solve for hit/commute times, there is also a “fundamental matrix” technique [10] for computing these quantities.

**Table 2:**
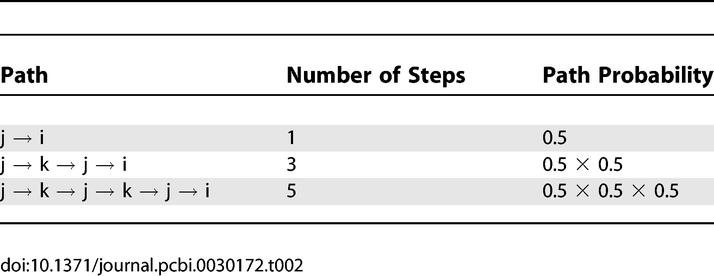
Enumerating *Some* of the Paths Connecting Node *j* with Node *i*

### Derivation of Equation 14.

The discussion below borrows from results in [12,13]. Deriving 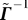 from ***Γ*** ^−1^ is a three-step process: (i) put together a matrix 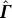 (size: *n* − 1 × *n*) from 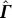 (size: *n* − 1 × *n* − 1) by appending a column vector ***p*** (size: *n* − 1 × 1):

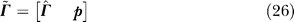
(ii) derive ***Γ*** (size: *n* × *n*) from 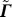 (size: *n* − 1 × *n*) by appending a row vector ***t*** *^T^* (size: 1 × *n* ):

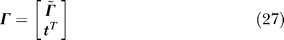
(iii) following the theory of generalized inverses [12] use 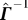 to express the inverse 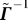 and then derive 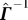 from ***Γ*** ^−1^.

The vectors ***p*** and ***t*** *^T^* are easy to derive because

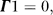
and

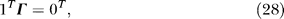
which implies that

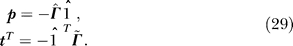
The generalized matrix inverse of 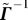 is given by

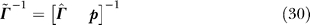


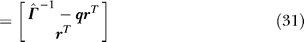
where

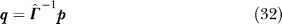


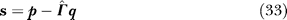
and

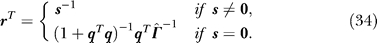
Substituting for ***p*** we obtain 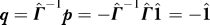 , and hence

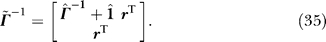


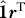
 is a rank-1 update to 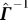 and ***r*** *^T^* is a row vector. So, we can tease out 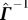 here by

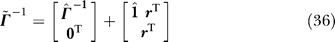
such that in component form

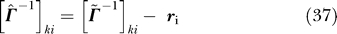


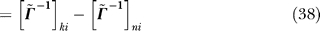
Now when we write

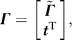
the effect of adding a row vector ***t*** *^T^* to 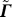 is similar to adding the column vector ***p*** to 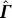 . So, as in Equation 38, the inverse of 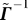 can be expressed as

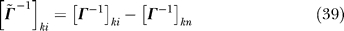
Putting the inverses in Equations 38 and 39 together, we obtain

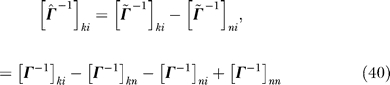
By substituting Equation 40 into Equation 13, we get

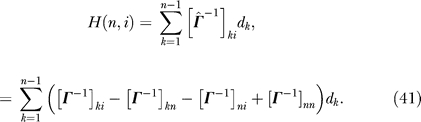
Using symmetry, the summation can be extended to *n* as in

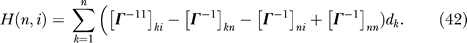
This derivation of hitting time to *n* ^th^ residue *v_n_* from *v_i_* was for convenience. For any arbitrary residue *v_j_*, the hitting time from residue *v_i_* will be

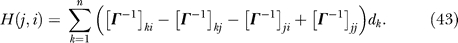



## Abbreviations

EN: elastic network
GNM: Gaussian Network Model
