# Correction: Signal Propagation in Proteins and Relation to Equilibrium Fluctuations

**DOI:** 10.1371/journal.pcbi.0030223

**Published:** 2007-10-26

**Authors:** Chakra Chennubhotla, Ivet Bahar

Correction for: Chennubhotla C, Bahar I (2007) Signal propagation in proteins and relation to equilibrium fluctuations. PLoS Comput Biol 3(9): e172. doi: 10.1371/journal.pcbi.0030172

Four mathematical expressions appeared incorrectly. The correct expressions follow.

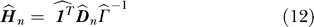


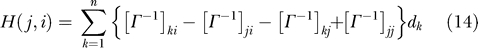
The hitting time expression Equation 14 involves three different types of contributions: a one-body term that depends on the destination node, 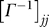 ; a two-body term that depends on the initial and final nodes, 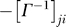 ; and a series of three-body terms that depend on intermediate nodes, in addition to the two end points, 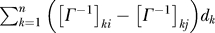 .

**Derivation of Equation 14.** The discussion below borrows from results in [12,13]. Deriving 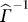 from 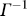 is a three-step process: …

